# The Epidemiology of Nomophobia and Insomnia Among Medical Students in Jeddah, Saudi Arabia

**DOI:** 10.7759/cureus.59927

**Published:** 2024-05-08

**Authors:** Sarah M Hussein, Sara S Alhwaiti, Ghada Almalki, Ghaya A Al Asadah, Raghad Mahdi, Cinderella Sami, Elaaf Dawood

**Affiliations:** 1 Clinical Sciences Department, Fakeeh College for Medical Sciences, Jeddah, SAU

**Keywords:** smartphone addiction, saudi arabia, medical students, insomnia, nomophobia

## Abstract

Background

Nomophobia and insomnia are common public health problems. The prevalence of moderate nomophobia is high among medical students in Saudi Arabia. Also, the prevalence of insomnia is high among the Saudi population. The relationship between nomophobia and insomnia is still unclear. So, the aim of this study is to investigate the epidemiology of nomophobia and insomnia among medical students in Jeddah, Saudi Arabia and to examine the relationship between them.

Subjects and methods

This is a cross-sectional study conducted among 157 medical students in Jeddah, Saudi Arabia. Data was collected by convenience sample and electronic questionnaire using Google form which was disseminated on social media. Nomophobia was assessed by a validated Nomophobia questionnaire (NMP-Q) and insomnia was assessed by Insomnia Severity Index (ISI).

Results

Most of the participants were females (78.3%) and the mean age of the study participants was 23.52 ± 2.61 years. The mean nomophobia score was 64.2 ± 19.5 and the mean insomnia score was 11.52 ± 4.31. A total of 58.6% of participants had a moderate level of nomophobia. In addition, 53.5% of participants had subthreshold insomnia and 28.7% had moderate insomnia. The study results did not reveal a significant relationship between nomophobia and insomnia. The academic year of the medical student is a significant risk factor for nomophobia.

Conclusion

The study findings suggest that a significant proportion of medical students in Jeddah, Saudi Arabia, suffer from nomophobia and insomnia. No significant association was found between nomophobia and insomnia. These findings highlight the urgent need to investigate factors that might contribute to these problems and developing interventions for nomophobia and insomnia among medical students.

## Introduction

Nomophobia is the feeling of fear of losing a mobile phone or being outside the network's coverage, thus not being able to call or receive communications. With the increase in the use of mobile phones, a new type of phobia has spread, known as “nomophobia” [1]. In a recent Saudi study, the prevalence of nomophobia (severe case) was recorded in one student among four students (22%) in Abha City, Saudi Arabia. Risk factors affecting grades of nomophobia are students’ age, older students who have a higher prevalence of severe nomophobia, students in college, with the highest prevalence of severe nomophobia among students in Applied Sciences College, and the lowest prevalence among medical students. Students who use smartphones for four hours or more daily and internet users have a higher prevalence of severe nomophobia [2].

Insomnia is a disorder that arises from an interruption, or low quality of sleep, which negatively affects the patient's mental and physical health. It can be defined as the complaint of difficulty initiating or maintaining sleep, not getting a comfortable sleep during the night, or getting up unusually early, and it affects the activity of the patient during the day. Its causes and treatments vary from person to person, according to his condition and circumstances [3].

Insomnia may occur alone or due to another problem. Disorders that can lead to insomnia include hyperthyroidism, heart failure, menopause, stress, chronic pain, heartburn, restless legs syndrome, and some medications, such as nicotine, caffeine, and alcohol. Other risk factors include night work and sleep apnea. Lifestyle change is usually the first treatment for insomnia. Cognitive behavioral therapy can be used to treat insomnia. While sleeping pills may help solve the problem, it has major problems as it is linked to addiction and dementia. Medications for more than four or five weeks are not recommended. The efficacy and safety of alternative medicine is not yet clear [3,4].

Saudi population showed a high prevalence rate of insomnia whereas a study conducted in Riyadh, Saudi Arabia, reported that the prevalence rate of insomnia among the Saudi population is 77.7% [5].

A Bahrain cross-sectional study conducted on 549 young adult populations demonstrated the association between nomophobia and insomnia. The study showed that 21% of participants had severe nomophobia, and 81 (14%) had clinical insomnia. A positive pair-wise linear correlation was observed between NMP-Q (Nomophobia Questionnaire) and ISI (Insomnia Severity Index) r=0.63, P = 0.001. Age, sex, BMI, and mobile phone screen size showed no association with the NMP-Q [6].

One of the most important reasons for fear of losing a mobile phone is the sign that addiction affects college students more than others; it affects the daily life of the individual. Nomophobia is a common public health problem that still needs more exploration; the relationship between nomophobia and insomnia is still unclear. Therefore, we conducted this study to assess the epidemiology of nomophobia and insomnia among medical students and investigate the association between them. The score and levels of nomophobia were assessed by using the NMP-Q, and the score and levels of insomnia by ISI, their risk factors, and the relationship between them. Therefore, this study determined the prevalence of nomophobia and insomnia among medical students in Jeddah, Saudi Arabia. In addition, it addressed factors that might associated with higher levels of nomophobia or insomnia among medical students and examined if there is an association between nomophobia and insomnia among medical students.

## Materials and methods

Study design

The current study was cross-sectional, to assess the epidemiology of nomophobia and insomnia among medical students and the relationship between nomophobia and insomnia.

Setting and population

All medical students (female-male) study in Jeddah, Saudi Arabia were eligible to participate in the study.

Sample size

The sample size was calculated based on the correlation between nomophobia and insomnia (r=0.25) [7]. So, the calculated sample size is 123 participants. However, we raised this sample size to 157 participants to overcome the non-response and increase the generalizability.

Sampling technique

It was a convenience sample, the research co-authors were medical students who disseminated the electronic questionnaire using Google form on social media such as WhatsApp and Telegram asking the medical students to participate in the study and fill out the questionnaire.

Study variables

1. Nomophobia which is the feeling of fear of losing a mobile phone or being outside the network's coverage.

2. Insomnia is a disorder, interruption, or low quality of sleep that negatively affects the patient's mental and physical health.

Data collection tool

A self-administered questionnaire includes:

(1) Socio-demographic such as age, gender, and academic year.

(2) Assessing the nomophobia by using the NMP-Q. This questionnaire was developed and validated by Yildirim and Correia (2015) [8]. It consists of 20 items on a Likert-type scale from 1 to 7; a score of 1 means strongly disagree yup to score of 7 means totally agree. The four dimensions of NMP-Q are divided into an inability to retrieve information (1-4 items), giving up convenience (5-9 items), inability to communicate (10-15 items), and losing connectedness (16-20 items). The scores vary between 20 and 140 points. Scores up to 20 indicate the absence of nomophobia, 21 to <60 indicates mild, 60 to <100 represents moderate, and 100≤ indicates severe nomophobia level. The Cronbach’s Alpha of NMP-Q is 0.88. So, it is a reliable tool.

Examples of the items of NMP-Q include asking if you feel uncomfortable without constant access to information through a smartphone, or annoyed if you cannot look information up on your smartphone when you want to do so. Being unable to get the news (e.g., happenings, weather, etc.) on your smartphone would make you nervous, and running out of battery in your smartphone would scare you; if you were to run out of credits or hit your monthly data limit, you would panic; if you could not check your smartphone for a while, you would feel a desire to check it; if you did not have your smartphone with you, you would feel nervous because you would not be able to receive text messages and calls; If you did not have your smartphone with you, you would feel weird because you would not know what to do, etc.

(3) Assessment of insomnia: to assess the severity of insomnia, the ISI was used. It included seven items. Every item is rated on a 0 to 4 scale. It is composed of: 

· Difficulty falling asleep

· Difficulty staying asleep

· Problems waking up too early

· How SATISFIED/DISSATISFIED are you with your CURRENT sleep pattern?

· How NOTICEABLE to others do you think your sleep problem is in terms of impairing the quality of your life?

· How WORRIED/DISTRESSED are you about your current sleep problem?

· To what extent do you consider your sleep problem to INTERFERE with your daily functioning (e.g. daytime fatigue, mood, ability to function at work/daily chores, concentration, memory, mood, etc.) CURRENTLY?

The total score ranges from 0 to 28. A higher score indicates more severe insomnia symptoms. A total score of ≥8 is identified as having symptoms of insomnia. The ISI is a reliable measure to evaluate perceived sleep difficulties (Cronbach’s alpha = 0.70-0.78) and a valid measure to detect changes in perceived sleep difficulties with treatment [9].

Data analysis

All data collected was coded by Excel, and statistical analysis was performed by using the Statistical Package for the Social Sciences (IBM SPSS Statistics for Windows, IBM Corp., Version 25.0, Armonk, NY). The descriptive statistics of sex, academic year, nomophobia, and insomnia levels were described as percentages. Levels of nomophobia were expressed as a pie chart and levels of insomnia were displayed as bar charts. The quantitative variables such as age, nomophobia, and insomnia scores were expressed as mean and standard deviation. The bivariate correlation between nomophobia and insomnia scores was conducted using Pearson correlation after assessing normality of variables, risk factors of nomophobia and insomnia were tested by using Fisher's Exact Test.

Ethical considerations

Informed consent was present at the beginning of the questionnaire, which described the aim of the study, and researchers’ information, and ensured the confidentiality and anonymity of participants. All participants provided informed consent before participation. Any participants could not start to fill out the questionnaire until agreed to participate at the end of the informed consent. The Institutional Review Board of Dr. Soliman Fakeeh Hospital, Jeddah, Saudi Arabia approved this study (Approval No: 383/IRB/2022). The study followed the principles of the Declaration of Helsinki, 2013. Confidentiality was preserved by keeping the participants' data secured and protected, the principal investigator was the only one who dealt with the data. Anonymity was achieved as the participants did not reveal any data related to their identity.

## Results

Table 1 shows a total of 157 individuals who were included in the final analysis, 123 of the participants were females (78.3%), the mean age of the study participants was 23.52 ± 2.61 years, the highest percentage of participants (33.8%) was in the sixth year (53 students), for nomophobia; the mean NMP-Q was 64.2 ± 19.5 ranged from 20 to 115. For the insomnia score, the mean score of ISI was 11.52 ± 4.31 and ranged from 4 to 26.

**Table 1:** Personal data of participants (n=157) The sex and academic year data have been represented as N and %. Age, nomophobia score, and insomnia score have been represented as mean ± SD and range.

Variable	N	%
Sex		
Male	34	21.7
Female	123	78.3
Age mean ± SD	23.52 ± 2.61	
Range	17-31	
Academic Year		
First	11	7
Second	11	7
Third	12	7.6
Fourth	22	14
Fifth	48	30.6
Sixth	53	33.8
Nomophobia score mean ± SD	64.2 ± 19.5	
Range	20-115	
Insomnia score mean + SD	11.52 ± 4.31	
Range	4-26	

**Figure 1:**
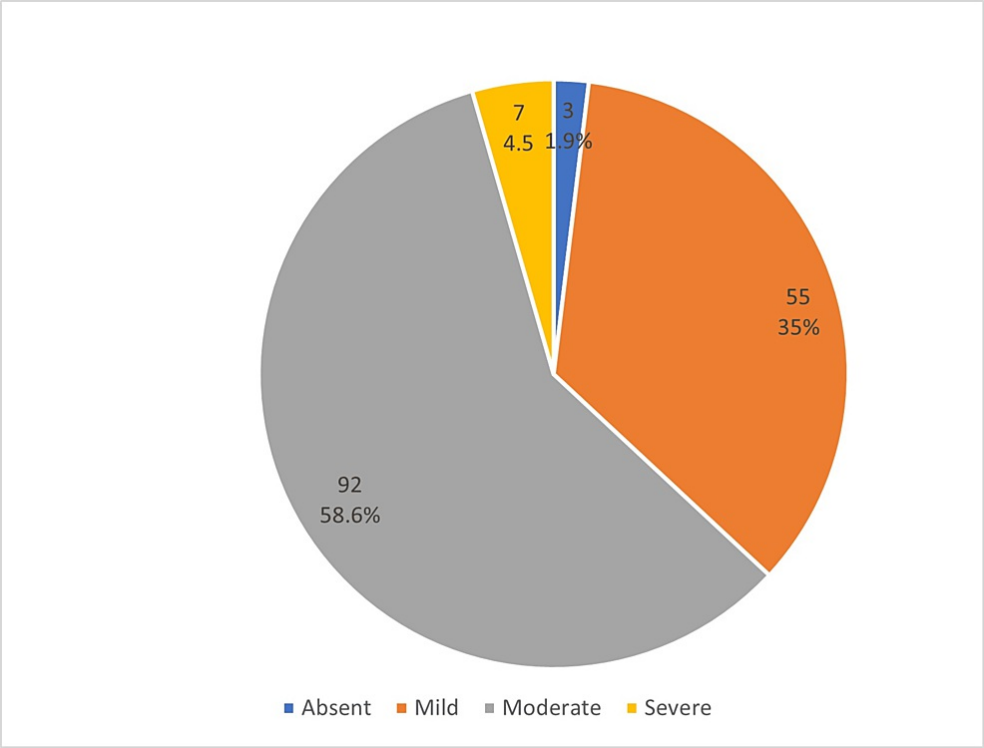
Nomophobia level among participants (n=157) The data have been represented as N and %.

Regarding the severity of nomophobia, as seen in Figure 1, three students (1.9%) did not have nomophobia, a total of 55 students (35.0%) had a mild level of nomophobia, 92 students (58.6%) had a moderate level of nomophobia and only seven students (4.5%) had severe nomophobia.

**Figure 2:**
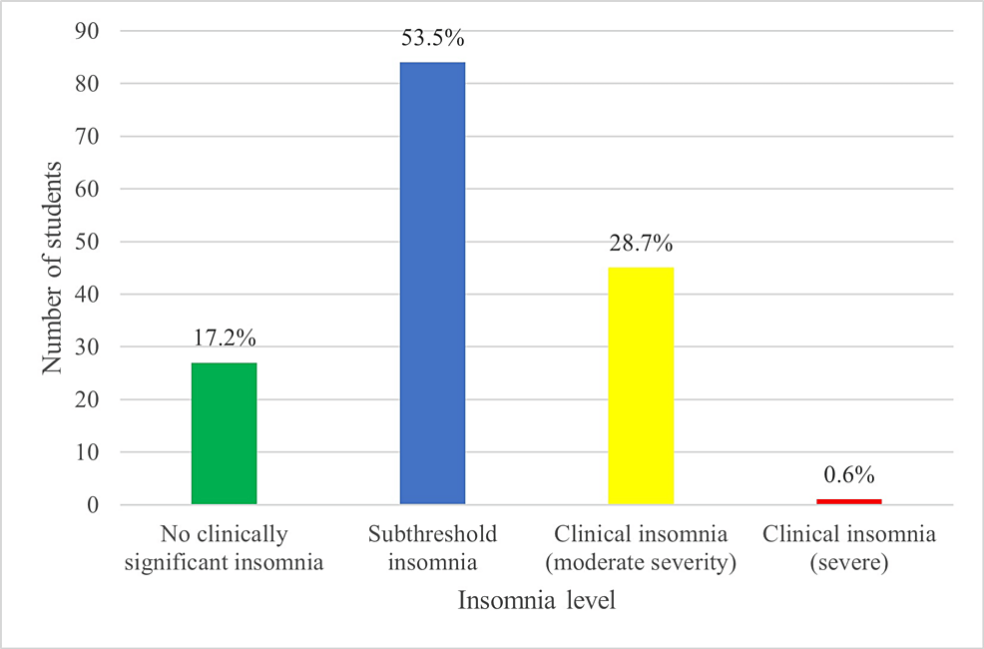
Insomnia level among participants (n=157) The data have been represented as N and %.

**Figure 3:**
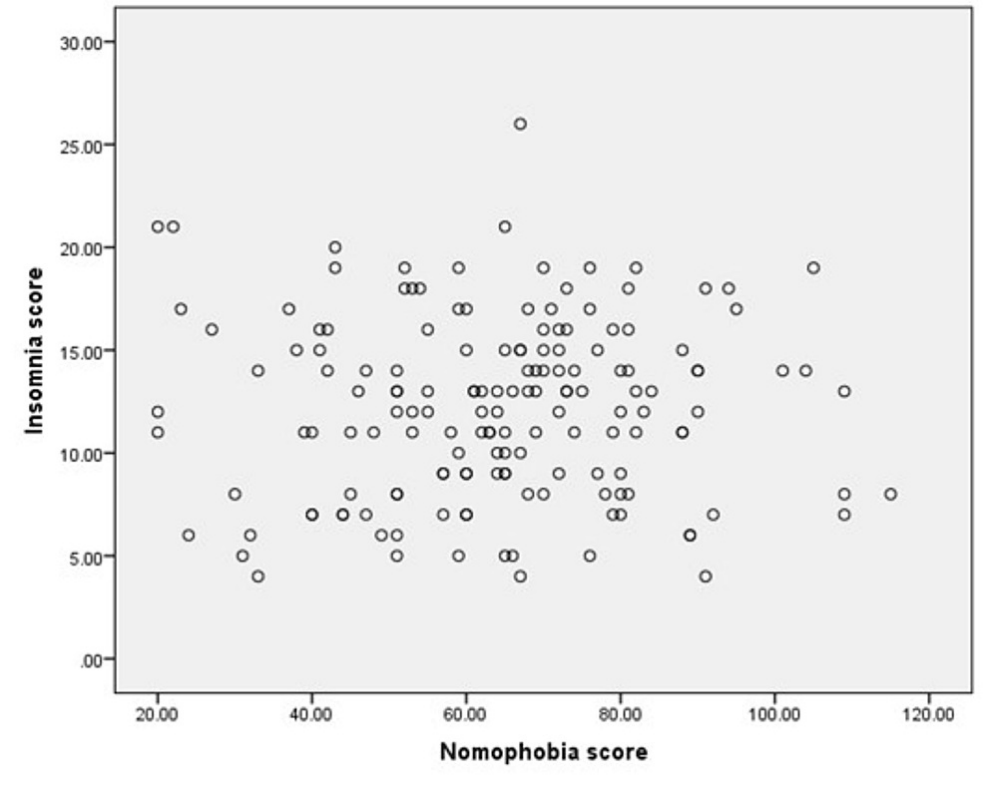
Association between nomophobia (NMP-Q score) and insomnia (ISI score) among participants (n=157) r =0.005, p=0.951, NMP-Q: Nomophobia questionnaire; ISI: Insomnia Severity Index

Figure 2 shows that approximately 27 students (17%) of the participants did not have clinically significant insomnia, 84 students (53.5%) had subthreshold insomnia and 45 students (28.7% of participants) had moderate insomnia. However, only one student (0.6%) had severe insomnia. Figure 3 shows that the correlation between insomnia and nomophobia scores was very weak, non-significance correlation (r =0.005, p=0.951).

Regarding factors level of nomophobia, 19 male students (55.9% of male participants) and 73 female students (59.3% of female participants) had moderate nomophobia. However, sex is not a statistically significant risk factor for nomophobia. With regards to age group, seven students aged less than 20 years had moderate nomophobia (58.3%), and 64 students aged from 20 to 25 years also had moderate nomophobia (56.6%). However, 21 students aged more than 25 years representing 65.6% of students in this age group had moderate nomophobia. However, age is not considered a statistically significant risk factor. The significant risk factor was the academic year, where six students in the first year (54.5%) had moderate nomophobia, in comparison to nine students in the second year (81.8%). Eight students in the third year (66.7%) had mild nomophobia, and four students in the fourth year had severe nomophobia (18.2%). With respect to students in years five and six, 30 students in year 5 (62.5%) and 34 students in year six (64.2%) had moderate nomophobia. Although 26 students (57.8%) with clinical insomnia (moderate severity) had moderate nomophobia, insomnia level does not statistically affect nomophobia (Table 2).

**Table 2:** Factors affecting the level of nomophobia among participants (n=157) The data have been represented as N and %. ^a^ Fisher's Exact Test. * Statistically significant at p < 0.05.

Personal characteristics	Nomophobia level				p-value
	Absent (n=3)	Mild (n=55)	Moderate (n=92)	Severe (n=7)	
	N (%)	N (%)	N (%)	N (%)	
Sex					0.382^a^
Males	1 (2.9)	11 (32.4)	19 (55.9)	3 (8.8)	
Females	2 (1.6)	44 (35.8)	73 (59.3)	4 (3.3)	
Age Group					0.684^a^
<20 years	1 (8.3)	4 (33.3)	7 (58.3)	0 (0.0)	
20-25 years	2 (1.8)	41 (36.3)	64 (56.6)	6 (5.3)	
>25 years	0 (0.0)	10 (31.3)	21 (65.6)	1 (3.1)	
Academic Year					0.045*^ a^
First	1 (9.1)	4 (36.4)	6 (54.5)	0 (0.0)	
Second	0 (0.0)	2 (18.2)	9 (81.8)	0 (0.0)	
Third	1 (8.3)	8 (66.7)	3 (25.0)	0 (0.0)	
Fourth	0 (0.0)	8 (36.4)	10 (45.5)	4 (18.2)	
Fifth	1 (2.1)	16 (33.3)	30 (62.5)	1 (2.1)	
Sixth	0 (0.0)	17 (32.1)	34 (64.2)	2 (3.8)	
Insomnia Level					0.498^a^
No clinically significant insomnia	0 (0.0)	14 (51.9)	12 (44.4)	1 (3.7)	
Subthreshold insomnia	2 (2.4)	24 (28.6)	53 (643.1)	5 (6.0)	
Clinical insomnia (moderate severity)	1 (2.2)	17 (37.8)	26 (57.8)	1 (2.2)	
Clinical insomnia (severe)	0 (0.0)	0 (0.0)	1 (100)	0 (0.0)	

Regarding factors affecting insomnia, as seen in Table 3, 17 male students (50% of males) and 67 female students (54.5% of females) had subthreshold insomnia. However, sex is not a statistically significant risk factor for insomnia. The age of participants, academic year, and level of nomophobia were also insignificant risk factors for insomnia.

**Table 3:** Factors affecting the level of insomnia among participants (n=157) The data have been represented as N and %. ^a^ Fisher's Exact Test. The p-value is considered significant if p<0.05.

Personal characteristics	Insomnia level				p-value
	No clinically significant insomnia (n=27)	Subthreshold insomnia (n=84)	Clinical insomnia (moderate severity) (n=45)	Clinical insomnia (severe) (n=1)	
	N (%)	N (%)	N (%)	N (%)	
Sex					0.365^a^
Males	5 (14.7)	17 (50.0)	11 (32.4)	1 (2.9)	
Females	22 (17.9)	67 (54.5)	34 (27.6)	0 (0.0)	
Age					0.442^a^
<20 years	3 (25.0)	6 (50.0)	3 (25.0)	0 (0.0)	
20-25 years	20 (17.7)	64 (56.6)	28 (24.8)	1 (1.9)	
>25 years	4 (12.5)	14 (43.8)	14 (43.8)	0 (0.0)	
Academic Year					0.419^a^
First	3 (27.3)	4 (36.4)	4 (36.4)	0 (0.0)	
Second	5 (45.5)	5 (45.5)	1 (9.1)	0 (0.0)	
Third	2 (16.7)	8 (66.7)	2 (16.7)	0 (0.0)	
Fourth	3 (13.6)	10 (45.5)	9 (40.9)	0 (0.0)	
Fifth	7 (14.6)	29 (60.4)	12 (25.0)	0 (0.0)	
Sixth	7 (13.2)	28 (52.8)	17 (32.1)	1 (1.9)	
Nomophobia Level					0.498^a^
Absent	0 (0.0)	2 (66.7)	1 (33.3)	0 (0.0)	
Mild	14 (25.5)	24 (43.6)	17 (30.9)	0 (0.0)	
Moderate	12 (13.0)	53 (57.6)	26 (28.3)	1 (1.1)	
Severe	1 (12.3)	5 (71.4)	1 (14.3)	0 (0.0)	

## Discussion

The current study sought to investigate the epidemiology of nomophobia and insomnia among medical students in Jeddah, Saudi Arabia. It also examined the relationship between nomophobia and insomnia.

With regard to the prevalence and severity of both nomophobia and insomnia, the current study found that a majority of participants (58.6%) had moderate levels of nomophobia. This finding corresponds with a previous study conducted in Saudi Arabia which found a 49% prevalence of moderate nomophobia among Saudi medical students [10]. Regarding insomnia, the current study findings revealed that 53.5% of the participants had subthreshold insomnia, 28.7% had moderate insomnia, and only 0.6% had severe insomnia. These results align with those of a prior study carried out in Jordan, which indicated that 44.3% of medical students had subthreshold insomnia and 21.4% experienced moderate insomnia [11].

Nomophobia has been associated with various psychological and behavioral health-related issues, including insomnia. Our study found a weak and non-significant relationship between nomophobia and insomnia scores among the participants. A study conducted in Bahrain found a strong positive correlation between nomophobia and insomnia among the general population, with higher levels of nomophobia being associated with higher odds of severe insomnia [6]. Similarly, a systematic review and meta-analysis revealed a significant correlation between nomophobia and insomnia symptoms [12]. Furthermore, another study in Saudi Arabia conducted on ESports players found an association between nomophobia and insomnia among Saudi adults [7]. This variation in the findings may be explained by different age groups and characteristics of the studied participants. In addition to a wide range of co-existing factors that may affect this relationship.

However, not all studies have found a direct relationship between nomophobia and insomnia. A study in Lebanon found that while higher anxiety and insomnia were significantly associated with severe nomophobia, nomophobia was unrelated to insomnia [13]. Another study on young adults found that nomophobia was associated with symptoms of anxiety although it was unrelated to insomnia [14].

Whereas numerous research has revealed a strong correlation between nomophobia and insomnia, others have found no clear relationship. Age, smartphone and social media addiction, and psychiatric problems all appear to have an impact on the association between nomophobia and insomnia, according to the studies. More research is needed to better understand nomophobia's origins and causes, as well as its relationship to insomnia.

Despite the absence of a significant link between nomophobia and insomnia, the current study's findings emphasize the importance of addressing both nomophobia and insomnia among medical students. Nomophobia, even at moderate levels, may adversely influence social interactions, educational performance, and overall well-being [15,16]. Similarly, insomnia has been associated with a variety of negative consequences, including poor academic performance, and an increased risk of physical and mental health issues [17,18].

Our study findings revealed no significant relationship between sex and nomophobia or insomnia levels. This is consistent with a recent study carried out in Turkey, which revealed no statistically significant difference between sex and nomophobia levels, while women had greater rates of nomophobia than men [19]. These findings contradict several previous studies. For example, a study of deaf and hard-of-hearing (DHH) children in Saudi Arabia discovered that nomophobia was more common in female DHH youth than in males [20]. Similarly, in a study of undergraduate students in Pakistan, women reported greater levels of nomophobia compared to men [21].

The discrepancies could be attributed to differences in age and general characteristics of the studied population. In addition to cultural or societal differences among the countries in which the research was conducted. Lastly, our findings shed light on the prevalence and severity of nomophobia and insomnia among medical students in Jeddah, Saudi Arabia, laying the groundwork for future research and interventions aimed at addressing these issues in this particular population.

Limitations

Nomophobia is one of the new emerging problems that affect a large sector of the population. Also, insomnia is one of the problems which has adverse effects on well-being. So, assessing these problems in medical students is crucial because this young age group is highly opposed to the adverse effects of these problems. This study has several strengths, one of these strengths is choosing the emerging problems that can affect physical, mental, and social well-being. Another strength point is using valid and reliable instruments such as NMP-Q and ISI questionnaires. Calculation of the sample size and enrolling adequate sample size enhance the reliability of the results. However, the study has some limitations. For instance, its cross-sectional design provides only a snapshot of the epidemiology of nomophobia, insomnia, and their relationship, making it impossible to establish causality or determine the temporal pattern. Longitudinal and follow-up studies are necessary to provide stronger evidence by tracking these relationships over time. Furthermore, the convenience sampling method used, which focused on medical students in Jeddah, Saudi Arabia, has limitations due to potential selection bias and restrictions on generalizability to larger populations. Future studies should use more diverse and representative sampling methods to improve external validity. In addition, using self-report questionnaires to assess nomophobia and insomnia introduces biases and errors such as response and recall biases, as well as social desirability bias, which reduces data accuracy and reliability. Lastly, the multifactorial nature of these problems subjects the results to the effect of confounders. In our study, we could not address these confounders. So, it is recommended in future research to examine these confounders in more detail.

Future studies that include objective measures or clinical assessments may help to mitigate these issues. Moreover, the study's subjective definitions of nomophobia and insomnia may result in differences in participant interpretation and reporting. Future research could benefit from greater precision in operationalizing these definitions, which could improve data collection consistency.

## Conclusions

The study findings suggest that a significant proportion of medical students in Jeddah, Saudi Arabia, suffer from nomophobia and insomnia. A total of 58.6% of participants have moderate levels of nomophobia and 53.5% have subthreshold insomnia. The significant risk factor for nomophobia was the academic year of the medical student. However, sex, age and insomnia were insignificant risk factors. For insomnia, among the studied factors, the study did not reveal statistically significant factors that can affect insomnia. In addition, no significant association was found between nomophobia and insomnia scores among studied participants. These findings highlight the urgent need to investigate more potential factors that might contribute to these issues and developing and implementing tailored interventions for nomophobia and insomnia among medical students.
